# Relationship between Dislocation Density and Antibacterial Activity of Cryo-Rolled and Cold-Rolled Copper

**DOI:** 10.3390/ma12020200

**Published:** 2019-01-09

**Authors:** Vinod Parmar, Kandarp Changela, B. Srinivas, Manimuthu Mani Sankar, Sujata Mohanty, S. K. Panigrahi, K. Hariharan, Dinesh Kalyanasundaram

**Affiliations:** 1 Centre for Biomedical Engineering, Indian Institute of Technology Delhi, New Delhi 110016, India; vinodparmar.9876@gmail.com; 2 Department of Physics, Indian Institute of Technology Delhi, New Delhi 110016, India; 3 Department of Mechanical Engineering, Indian Institute of Technology Delhi, New Delhi 110016, India; kandarp.changela06@gmail.com; 4 Department of Mechanical Engineering, Indian Institute of Technology Madras, Chennai 600036, India; srinivasbehera108@gmail.com (B.S.); skpanigrahi@iitm.ac.in (S.K.P.); hariharan@iitm.ac.in (K.H.); 5 Stem Cell Research Facility, All India Institute of Medical Sciences, New Delhi 110029, India; sankar.mm@gmail.com (M.M.S.); drmohantysujata@gmail.com (S.M.); 6 Department of Biomedical Engineering, All India Institute of Medical Sciences, New Delhi 110029, India

**Keywords:** defects, nano-crystalline, cryo-rolling, antibacterial, dislocation, copper, leaching, nanostructuring

## Abstract

In the present work, cold rolling and cryo-rolling were performed on 99% commercially pure copper substrates. Both cold and cryo-rolling processes caused severe plastic deformation that led to an increase in dislocation density by 14× and 28× respectively, as compared to the pristine material. Increases in average tensile strengths, by 75% (488 MPa) and 150% (698 MPa), were observed in the two rolled materials as the result of the enhancement in dislocation density. In addition to strength, enhanced antibacterial property of cryo-rolled copper was observed in comparison to cold rolled and pristine copper. Initial adhesion and subsequent proliferation of bio-film forming Gram-positive bacteria *Staphylococcus aureus* was reduced by 66% and 100% respectively for cryo-rolled copper. Approximately 55% protein leakage, as well as ethidium bromide (EtBr) uptake, were observed confirming rupture of cell membrane of *S. aureus.* Inductively coupled plasma-mass spectroscopy reveals higher leaching of elemental copper in nutrient broth media from the cryo-rolled copper. Detailed investigations showed that increased dislocation led to leaching of copper ions that caused damage to the bacterial cell wall and consequently killing of bacterial cells. Cryo-rolling enhanced both strength, as well as antibacterial activity, due to the presence of dislocations.

## 1. Introduction

Infection by bacteria via domestic contact is a concern in public areas. Commonly used materials, such as stainless steel, are ineffective in controlling the proliferation of bacteria. In addition to antibacterial activity, the metals used in hospital environments should be non-cytotoxic to human cells. Copper possesses the distinct advantages of being antibacterial and having a non-cytotoxic property [1]. Copper kills bacteria by a mechanism termed as “contact killing”, wherein bacterial cells are damaged upon contact with the surface of copper. Release of copper ions from the surface of plates is essential for the contact killing mechanism. Other factors, such as corrosion, surface texture, ingredient/impurity elements in copper plates and microbes present influence contact killing. Although the exact mechanism of contact killing is not yet clear, the most likely phenomenon during contact killing is as follows: Severe damage occurs to the cell envelope and cytoplasmic membrane in the first stage; and damage to intercellular components, including the DNA of micro-organisms occurs in the second stage. In the case of a dry surface, the aqueous phase surrounding the bacteria enables the influx of copper ions. Under ambient conditions, copper oxidizes to Cu_2_O and CuO; and these oxides are soluble in aqueous media. After dissolution, Cu^1+^/Cu^2+^ ions are released by the oxides. The aforementioned copper ions damage the cell wall and hence copper shows antibacterial properties [2]. This antibacterial property makes it a preferred material for biomedical applications [3]. Apart from possessing excellent antibacterial properties [4], copper is non-toxic to human cells and tissues, making it an ideal choice for such applications. However, copper possesses less strength when compared to the other traditionally-used biocompatible stainless steel–SS316L (yield strength of pure copper is typically 70 MPa that is approximately one third that of SS316L) [5,6].

The biomedical applications of copper are limited, primarily due to its low strength, which can be improved by severe plastic deformation (SPD) [7,8,9]. The strength of copper can also be improved by stoichiometric alloying with appropriate elements. Ultrafine grain refinement through SPD is successful in achieving an excellent combination of mechanical properties when compared to alloying [9,10]. SPD is known to produce materials with nano-crystalline grain structures. Most of the SPD techniques, such as high-pressure torsion (HPT), accumulative rolling bonding, equal channel angular pressing, dynamic plastic deformation and severe cold rolling, subject the material of choice to very large plastic strain through high hydrostatic stress [11]. Conventional plastic deformation processes, such as cold rolling, restrict the maximum plastic strain that can be applied to a material before failure and thus limit the minimum grain size that can be produced. In the case of severe plastic deformation, a large plastic strain leads to ultra-fine grain structures, resulting in materials with new sets of mechanical properties, especially very high strength and reasonable ductility [12].

Among the different SPD processes, cryo-rolling has a distinct advantage for producing bulk nanostructured materials without altering the conventional rolling setup. The process involves immersion of the substrate in liquid nitrogen for a predefined time interval followed by immediate rolling. The cyclic procedure of immersion in liquid nitrogen and rolling is repeated until the desired thickness is achieved. The time interval should be adequate to achieve thermal equilibrium of the entire substrate at cryogenic temperatures (approximately 77 K) [13]. The cyclic cryogenic treatment suppresses dynamic recovery, thereby enhancing the strength of the substrate by extending the stage-II (athermal hardening) strain hardening regime [14,15,16]. In addition to strength, other mechanical properties, such as wear resistance and fatigue resistance, are also improved by SPD processes [11].

Nanostructured copper is also known for its antibacterial property [17]. *Staphylococcus aureus* (*S. aureus*) is a Gram-positive, aerobic, and pathogenic organism of 0.5–1.5 µm, causing skin infections, pneumonia, endocarditis, osteomyelitis, and finally leading to sepsis. The ability of *S. aureus* to adhere to the extracellular matrix and plasma proteins on the surface of biomaterials or implants is a key factor for its dissemination on surgical implants and pathogenesis in surgical procedure related infections. *S. aureus* propagates rapidly and has a repertoire of pathogenic mechanisms to evade the host defense system and cause infection. It is also noted that not all *S. aureus* strains have similar abilities of producing adhesions or toxins. They may also differ in biofilm production and virulence factors [18,19]. *S. aureus* is also responsive to a majority of infections associated with medical devices, ranging from scissors to replacement heart valves [19]. While the mechanical behavior of cryo-rolled copper has been reported previously [13], its effect on pathogenic bacteria, such as Gram-positive *Staphylococcus aureus* (*S. aureus*), has not been reported yet. This is the first work to report the correlation between defects post nano-structuring to the antibacterial effect of cryo-rolled copper on pathogenic bacteria, such as Gram-positive *Staphylococcus aureus* (*S. aureus*). The objective of the present work is to investigate the mechanism responsible for the process induced enhancement of antibacterial properties in copper. Experimental observations and correlations with metallurgical characterization in cold rolled and cryo-rolled copper were used to explain the phenomenon.

## 2. Materials and Methods

### 2.1. Material Processing

Six-millimeter thick, 99.9% pure copper substrates were subjected to both cold rolling and cryo-rolling in a conventional four high rolling mill (Make: Vaid Engineering Industries, New Delhi, India). The working roller diameter was 142 mm, while the angular velocity of the roller was approximately π rad/s (32 rpm). Commercially pure (99.9%) copper substrates with dimensions of 150 mm × 100 mm × 6 mm (thickness) were used as the raw material (initial).

**Figure 1:**
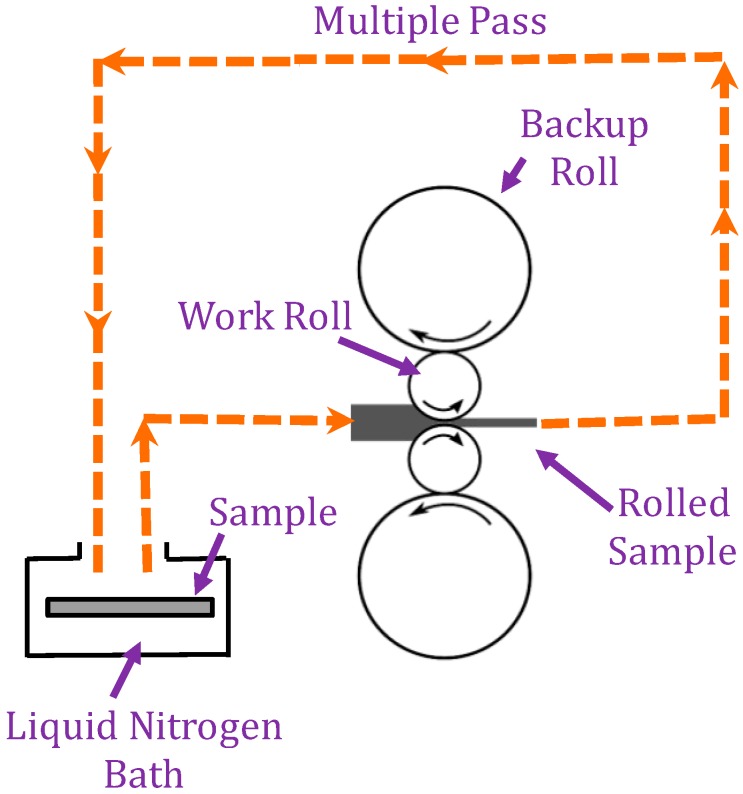
Schematic of the cryo-rolling operation.

One set of copper substrates was cold rolled to 1 mm at room temperature with a 5% reduction per pass. The second set of copper substrates was rolled after immersion in a liquid nitrogen bath (maintained at 77 K) for 15 min. The experimental setup for cryo-rolling and cold rolling processes are shown in Figure 1. Due to cryogenic temperatures, only a 4.5% reduction per pass was set. The substrates were rolled immediately. Subsequent cryo-rolling passes were performed after intermittent immersion of the copper substrates in liquid nitrogen for five minutes, as shown in Figure 1. Strength, hardness and wettability properties were evaluated by mechanical tests, while the microstructures were studied by XRD and TEM.

### 2.2. Physical and Mechanical Characterization

X-ray diffraction studies (Ultima-IV, Rigaku, TX, USA) at a 1.54 Angstrom wavelength using Cu Kα radiation with a scan rate of 4° per minute, in bulk (for 2θ range of 10 to 80°), were performed on 10 mm × 10 mm copper coupons after careful metallographic polishing. The diffraction data were analyzed using PCPDF software and JCPDS database (JCPDS Ref No.: 04-836, 03-105, 78-2076). Contact angle measurements were performed using a DataPhysics OCA 15EC goniometer (DataPhysics Instruments GmbH, Germany). Two-microliter droplets of deionized (DI) water were dispensed on the surface of the coupons. After one minute of stabilization, the contact angles were measured. The micro hardness of the substrates was measured using a Vickers hardness tester (Model: RVM 50PC, Rockwell Testing Aids, India). Uniaxial tensile tests of cold-rolled and cryo-rolled samples were performed in an Universal Testing Machine (UTM) (Model: 5582, Capacity: 50 kN, Instron, USA) The specimens for tensile testing were profile cut using wire cut electric discharge machining in accordance with the ASTM-E8 standard. For TEM investigations, cryo-rolled and cold rolled copper were gently polished to obtain a foil measuring 80 µm. A blank of 3 mm in diameter was punched from the foil. The samples were further thinned by ion beam milling (PIPS II, Gaton Inc., Pleasanton, CA, USA) to obtain a transparent region for transmission of the beam. For this purpose, an ion milling machine operating at an accelerating voltage of 5 kV was used for 1.5 to 3 h. Finally, TEM analysis was carried out on a CM12 machine (Philips, Amsterdam, Netherlands) operating at an accelerating voltage of 120 kV.

### 2.3. Adhesion and Proliferation of S. Aureus Cells on Copper Coupons

Staphylococci are prime bacteria causing hospital-acquired infections and are well known to form biofilms on any metal surface that they come into contact with. In general, *S. aureus* is the most common bacterium responsible for surgical site infections in cardiac and orthopedic surgeries [20,21]. *S. aureus* expresses poly-β(1–6)-N-acetylglucosamine (PNAG) polysaccharide, a major constituent present in the biofilms of several Gram-positive and Gram-negative organisms [22]. Hence, *S. aureus* was used as a model bacterium in this study. Coupons, 5 mm × 10 mm in size, with respective thicknesses of the substrate were cleaned by bath sonication for 15 min, each, in acetone followed by deionized water. The coupons were air dried and sterilized by autoclaving at 121 °C for 40 min. *S. aureus* cell lines (*S. aureus*, subsp. aureus, ATCC 25923) were maintained on Muller Hinton agar (Hi-Media, India) under standard aerobic conditions at 37 °C. All bacterial experiments were performed under a biosafety level-2 hood.

#### 2.3.1. Bacterial Adhesion and Progression Assay

Sterilized pristine, cold rolled, and cryo-rolled copper coupons were placed in 24-well culture plates in triplicates. One-hundred-microliter inoculums of *S. aureus* suspensions of 105 CFU/mL concentration were placed on the surface of the copper coupons and incubated for 2 h at room temperature (~25 °C). After the incubation period, non-adherent *S. aureus* were gently washed twice using 1X phosphate buffer saline (PBS, Sigma Aldrich, USA) at pH 6.8. The copper coupons were placed into fresh 24 well plates and 1 mL of nutrient broth (Hi-Media, India) was added into the wells. Three sets of coupons were further incubated at 37 °C, each for time intervals of 0, 24 and 72 h.

#### 2.3.2. Bacterial Adhesion and Progression Assay

After the incubation period, copper coupons were removed from the respective wells and gently washed with sterile 1X PBS to remove non-adherent cells. The copper coupons were placed in glass tubes containing 1 mL of sterile DI water. The tubes were vortexed for 2 min and sonication for 15 min to obtain a homogenous bacterial cell suspension from the coupons. The obtained suspensions were serially diluted to seven-fold dilutions in 1X PBS (pH 6.8). A volume of 100 µL of the diluted suspension was spread plated on Muller-Hinton agar plates in triplicates. The agar plates were incubated at 37 °C for 24 h. Colony-forming units (CFUs) were enumerated and the results were expressed in CFU/mL. The bacterial reduction (BR) rate was calculated as the ratio of the difference in colonies on the test coupons (cold-rolled and cryo-rolled) to the number of colonies on pristine coupons at different time intervals.

The disruptive effect of copper on *S. aureus* was assessed by ethidium bromide (EtBr) uptake assay. *S. aureus* treated with 1% Triton X-100 was used as a positive control whereas cells with EtBr not exposed to copper substrates served as negative controls. Ten microliters of each suspension were examined under a fluorescence microscope. Inductively coupled plasma mass spectrometry (ICP-MS) was used to estimate the leaching of elemental copper from pristine, cold rolled and cryo-rolled copper coupons in the nutrient broth. Detailed information on the toxicity, other cellular studies and leaching of copper ions are given in the supplementary section.

## 3. Results and Discussion

The average micro-Vickers hardness of pristine, cold rolled, and cryo-rolled were 70 HV, 130 HV, and 180 HV respectively. The tensile strength of the pristine, cold rolled and cryo-rolled were 279 MPa, 488 MPa and 698 MPa respectively (as reported in the Supplementary Information, Figure S2). Cryo-rolling was performed in 39 passes while cold rolling was performed at 35 passes. It has been established that the slower rate of strain hardening rate also contributes to the increase in strength [23]. The crystalline size (*d*) is computed using Williamson-hall method [24] and is used to calculate dislocation density [25,26]. The peaks of Cu (111) and (220) broadened after cryo-rolling due to the combined effects of reduced crystallite size (*d*), increased residual strain (ε) and increased dislocation density (ρ) are reported in Table 1. The crystalline size (d) was calculated using the Williamson-Hall equation [27], as per Equation (1),

(1) $$d=\frac{k\lambda }{B\;\cos\theta -4\;\varepsilon \sin\theta }$$

where *ε* is micro-strain, *θ* is diffraction angle and *B* is peak broadening width. These parameters (*ε*, *θ*, and *B*) were determined by examining the XRD peak broadening using well-known XRD analysis software (PANalytical X’pert Highscore). To improve the accuracy of XRD analysis, careful filtration of unwanted instrumental broadening was done by analyzing the XRD peaks of a standard specimen using the deformed copper samples (cold-rolled and cryo-rolled). The dislocation density (ρ) was calculated using Equation (2) [25,28],

(2) $$\rho =\frac{2\sqrt{3}{\left(\varepsilon^{2}\right)}^{\frac{1}{2}}}{d\;b}$$

where *b* is the magnitude of Burgers vector. The increase in the Vickers hardness and the tensile strength is a result of smaller crystalline sizes and an increase in dislocation density (Table 1).

**Figure 2:**
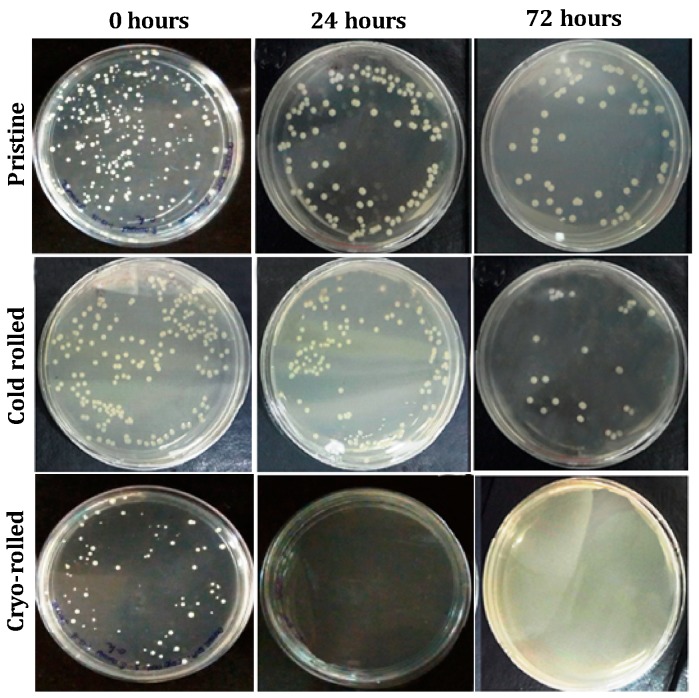
Viability of *S. aureus* retrieved from copper coupons: Agar plate pictures for 0, 24 and 72 h.

**Figure 3:**
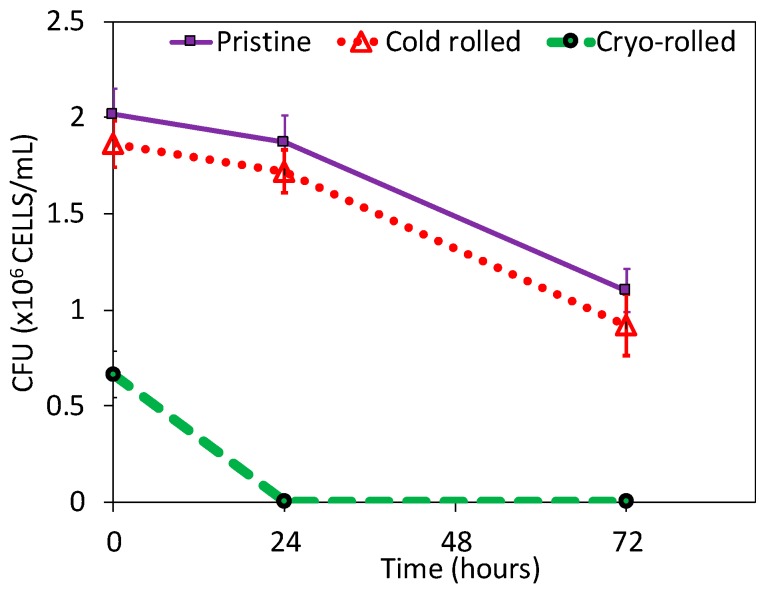
Colony forming units (CFU) of bacterial growth on pristine, cold rolled and cry-rolled surface.

**Figure 4:**
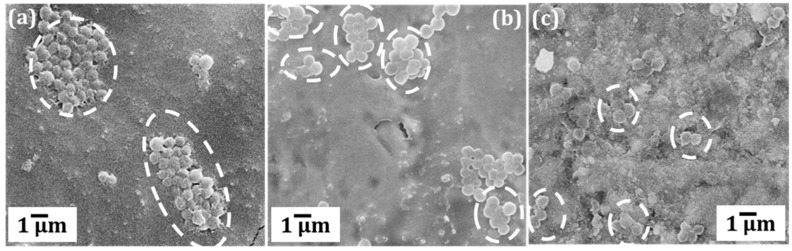
SEM micrographs of *S. aureus* colonies formed after 24 h over (**a**) pristine (**b**) cold rolled and (**c**) cryo-rolled copper coupons.

After the initial exposure, cryo-rolled coupons exhibited reduced adhesion compared to cold rolled and pristine coupons, although the contact angle (usually exhibits inverse correlation with adhesion) measured was lower (as shown in Figure S1 in the Supplementary Information). On further exposure for 24 and 72 h, cryo-rolled coupons exhibited antibacterial activity. The retrieved cells from the coupons were cultured on MacConkey^®^ agar plates for 24 h (Figure 2). However, both cold rolled and pristine copper did not inhibit adhesion and progression of *S. aureus*. The adhesion was reduced by 7.73% and 67.11%, respectively, in the cold-rolled and cryo-rolled samples during initial exposure. After the 24-h exposure, cryo-rolled coupons demonstrated a 100% reduction whereas cold-rolled coupons exhibited a mere 8.02% reduction. After 24 h growth, it was observed that *S. aureus* established a higher CFU on pristine than on cold-rolled or cryo-rolled copper coupons, as shown in Figure 3. The number of bacterial cells per colony decreased drastically for cryo-rolled copper, indicating significant impairing of bacterial cell growth and colony formation (Figure 4). Though *S. aureus* cells were observed on cryo-rolled copper coupons at 0 h, they were not viable, as seen in Figure 2.

*S. aureus* cells exposed to cryo-rolled coupons exhibited increased staining 4-fold of EtBr as compared to pristine and cold-rolled copper (Figure S4 in Supplementary Information). The mechanism responsible for this improvement is not been established thus far. On the other hand, studies on the biocompatibility of ultrafine-grained materials in recent years [29] suggest a better adhesion of cells, given the material’s wettability (from contact angle measurements) [30]. This conflicts with our present results. It is to be, however, clarified that the hydrophilicity confirmed via contact angle does not guarantee the chemical affinity between the cell wall and adhering surface.

Contact killing by Cu^1+^/Cu^2+^ ions is the widely-accepted mechanism for copper’s antibacterial property. Since the materials investigated differ only in their substructures (Table 1), it is pertinent to explore the influence of dislocation density on the ionization of copper. Ionization of copper, when in contact with aqueous medium, leads to the creation of static charge field around the dislocations [31]. Charged dislocations are generally surrounded by a cloud of charged vacancy defects ‘*q*’ [32]. The interaction between this charge (*q*) and dislocation can be correlated with the ease of Cu ion formation. The dislocation-charged cloud interaction energy (*E*_int_) [33,34] is expressed as:

(3) $$E_{\mathrm{int}}=E_{elas}+E_{Coul}=\frac{Gb}{3\pi }\frac{1+\upsilon }{1-\upsilon }\frac{y^{2}}{x^{2}+y^{2}}dv+\frac{-2q\lambda }{\varepsilon }\ln\left(r\right)$$

where *G* and *ν* are elastic constants, *ε* is the relative permittivity and *x* and *y* are the position vectors of the dislocation. The lower the *E*_int_, the easier is the formation of ions. Since the elastic constants in *E_elas_* are invariant, *E*_int_ differs only by the electrostatic coulomb interaction energy (*E_Coul_*). The charge, ‘*q*’ around the dislocation can be estimated using the Koehler equation [35].

(4) $$q=\left(\zeta kT/2e\right)Ka\frac{\partial z}{\partial s}$$

where ζ is the static charge constant value, *T* is the temperature *k* is the Boltzmann constant, *a* is the material constant, ‘*s*’ is the mean free path of dislocation and $\frac{\partial z}{\partial s}$ is the field potential gradient around the dislocation. To understand the ease of ion formation, it is necessary to relate the dislocation potential (*z*) to the dislocation density, as given by Pödör [36].

(5) $$z=\rho \;s\;b\frac{m_{e}}{h^{3}\zeta^{2}c^{2}}{\left[\beta^{4}{\left(1+\frac{4k^{2}}{\beta^{2}}\right)}^{1.5}\right]}^{-1}$$

where ρ is the dislocation density, *m_e_* represents electron effective mass, *c* is the lattice constant, and *β* is the inverse screening length. Among the three copper samples, ρ is the only variable in Equation (5), therefore:

(6) $$\frac{\partial z}{\partial s}=\phi \rho$$

where ϕ=z/ρ s. Substituting Equation (6) into Equation (4),

(7) q=[(ζkT/2e) Kaϕ]ρ

using Equation (7), *E_Coul_* can be expressed as

(8) $$E_{Coul}=-\rho \left(\frac{\zeta kTa\phi \lambda }{\varepsilon e}\ln\left(r\right)\right)$$

Since cryo-rolled material has the highest dislocation density (Table 1), the E_int_ of this material will be lower as per Equations (3), (7) and (8). This will result in higher extent of Cu ion formation (Cu^2+^ and Cu^+^) in cryo-rolled material leading to better antibacterial property. The antibacterial property of the three samples can be directly correlated to the dislocation density. The quantitative results of inductively coupled plasma mass spectrometry (ICP-MS) indicate that elemental copper leached out from pristine, cold rolled and cryo-rolled copper coupons in the amounts of 644.5, 975.9, and 1441.5 ppb/mm^2^ respectively, into the nutrient broth (Supplementary Information). The increased leaching of copper from cryo-rolled copper as compared to cold rolled and pristine supports the above discussion. The increased ion density of cryo-rolled copper, confirmed by leaching, has led to higher contact killing *S. aureus*. However, the cryo-rolled copper proved to be non-toxic to humans in a hemolysis experiment (as shown in Figure S5 in the Supplementary Information).

**Figure 5:**
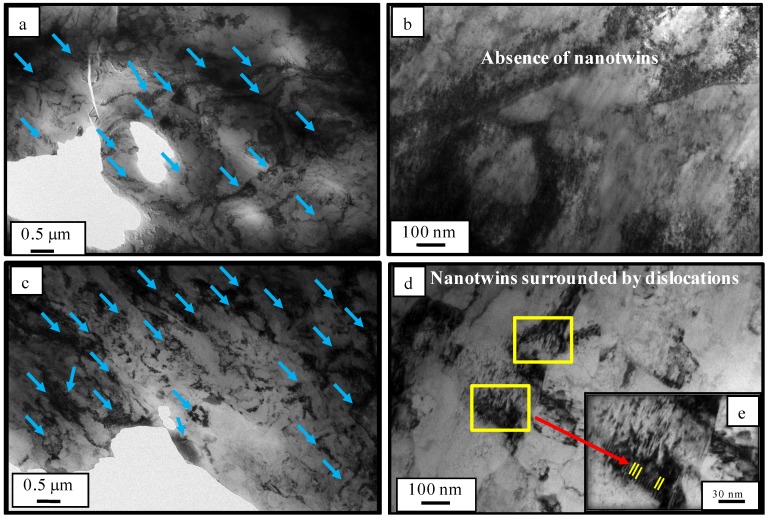
TEM of cold rolled copper (**a**,**b**) and cryo-rolled copper (**c**,**d**). (**e**) Magnified view (blue arrow mark represents dislocation tangles/clusters).

Both cryo-rolled and cold rolled materials were investigated for the sites of dislocations by transmission electron microscopy (TEM). In cold-rolled and cryo-rolled materials, the micrograph represents several sites with densely packed dislocation tangles/ clusters(Figure 5a,c). In the cryo-rolled material, dislocation tangle/clusters appear to be denser in Figure 5d, as compared to the cold rolled material shown in Figure 5b. In addition to the dense dislocation cluster, cryo-rolled materials also exhibit closely packed nanotwins surrounded by packets of dislocation Figure 5d,e. The reason for such nanotwin in cryo-rolled can be attributed to (i) low stacking fault energy of copper; (ii) cryogenic processing temperature; and (iii) movement of Shockley partials via cross slip. The nanotwins in the form of Shockley partials also contribute to the overall dislocation density of the cryo-rolled material.

On the basis of all of the above results, we conclude that the cryo-rolled copper contains denser dislocation clusters and nanotwins (as reported in Figure 5). The higher dislocation density results in enhanced leaching of copper ions due to direct effect of dislocation density on the charges. The enhancement of the leaching of copper ions was verified by the ICP-MS results. The leaching of copper ions helps in the disruption of the cell wall of *S. aureus* bacteria, the EtBr uptake assay and protein leakage results (Figure S3 in the Supplementary Information) shows significant evidences of damage of cell-wall of bacteria during interaction of copper surface with adhered bacterial species. Therefore, excessive leaching of copper ions from high strength cryo-rolled samples promotes contact killing of *S. aureus* and thus results into improved antibacterial property of the cryo-processed copper. Due to enhancement of antibacterial property of high strength cryo-rolled copper, initial adhesion and subsequent proliferation *S. aureus* were reduced by 66% and 100%, respectively, as shown in Figure 3.

## 4. Conclusions

In summary, it can be stated that the cryo-rolling process for strengthening also enhances the antibacterial property of copper. The dislocations in cryo-rolled copper are surrounded by positively-charged electrostatic clouds, that in-turn leads to an increased internal energy. In this first reported work, experimental observations on bacterial adhesion and cell wall disruption indicate that cryo-rolled copper is the most effective antibacterial material as compared to cold rolled and pristine copper. An in-depth investigation of enhanced antibacterial property of the cryo-rolled copper revealed that denser dislocations are induced by the cryo-rolling process which lead to release of excessive copper ions as compared to pristine copper. These copper ions damage the bacterial cell wall, resulting in the contact killing of bacterial cells and therefore the cryo-rolling process improves antibacterial property significantly. The major contribution of the present work is the relationship between dislocation density, leaching of copper ions and the antibacterial property.

## Figures and Tables

**Table 1:** Structural properties of the three copper samples.

S. No	Material Condition	Crystalline Size (*d*) (nm)	Microstrain (*ε*) (%)	$\mathrm{Dislocation}\;\mathrm{Density}/{\mathrm{mm}}^{2}\;(\rho )$ (× 10^18^)	Average Contact Angle (°)
1	Pristine	170.2	0.028	0.018	114.0
2	Cold rolled copper	72.4	0.117	0.278	96.3
3	Cryo-rolled copper	55.7	0.262	0.526	80.8

## Supplementary Materials

The following are available online at http://www.mdpi.com/1996-1944/12/2/200/s1, Figure S1: Apparent contact angle (a) pristine, (b) cold rolled and (c) cryo-rolled copper, Figure S2: Stress-strain curve depicting tensile test results of pristine, cold rolled, cryo-rolled 1, and cryo-rolled 2 copper substrates, Figure S3: Percentage leakage of protein exposed to copper coupons, Figure S4: EtBr uptake of *S. aureus* on (a) pristine, (b) cold rolled, (c) cryo-rolled copper site1, and (d) cryo-rolled copper site 2 coupons (100X magnification), Figure S5: Percentage hemolysis of RBCs exposed to copper coupons.

## References

[1] Alavi M., Karimi N. (2017). Characterization, antibacterial, total antioxidant, scavenging, reducing power and ion chelating activities of green synthesized silver, copper and titanium dioxide nanoparticles using Artemisia haussknechtii leaf extract. Artif. Cells Nanomed. Biotechnol..

[2] Hans M., Mathews S., Mücklich F., Solioz M. (2016). Physicochemical properties of copper important for its antibacterial activity and development of a unified model. Biointerphases.

[3] Champagne V.K., Helfritch D.J. (2014). Mainstreaming cold spray—Push for applications. Surf. Eng..

[4] Zhao D., Lu Y., Zeng X., Wang Z., Liu S., Wang T. (2017). Antifouling property of micro-arc oxidation coating incorporating Cu_2_O nanoparticles on Ti6Al4V. Surf. Eng..

[5] Krishnaiah A., Chakkingal U., Venugopal P. (2005). Applicability of the groove pressing technique for grain refinement in commercial purity copper. Mater. Sci. Eng. A.

[6] Bailat C., Gröschel F., Victoria M. (2000). Deformation modes of proton and neutron irradiated stainless steels. J. Nucl. Mater..

[7] Cordero Z.C., Knight B.E., Schuh C.A. (2016). Six decades of the Hall–Petch effect—A survey of grain-size strengthening studies on pure metals. Int. Mater. Rev..

[8] Yogesha K.K., Joshi A., Kumar N., Jayaganthan R. (2017). Effect of cryo groove rolling followed by warm rolling (CGW) on the mechanical properties of 5052 Al alloy. Mater. Manuf. Process..

[9] Satish D.R., Feyissa F., Kumar D.R. (2017). Cryorolling and warm forming of AA6061 aluminum alloy sheets. Mater. Manuf. Process..

[10] Nanda T., Kumar B.R., Sharma S., Singh V., Pandey O.P. (2017). Effect of thermal cycling process parameters on recrystallization kinetics for processing of fine-grained pure copper. Mater. Manuf. Process..

[11] Valiev R. (2004). Nanostructuring of metals by severe plastic deformation for advanced properties. Nat. Mater..

[12] Valiev R.Z., Estrin Y., Horita Z., Langdon T.G., Zechetbauer M.J., Zhu Y.T. (2006). Producing bulk ultrafine-grained materials by severe plastic deformation. JOM.

[13] Anand G., Barai K., Madhavan R., Chattopadhyay P.P. (2015). Evolution of annealing texture in cryo-rolled copper. Mater. Sci. Eng. A.

[14] Kocks U.F., Mecking H. (2003). Physics and phenomenology of strain hardening: The FCC case. Prog. Mater. Sci..

[15] Srinivas B., Dhal A., Panigrahi S.K. (2017). A mathematical prediction model to establish the role of stacking fault energy on the cryo-deformation behavior of FCC materials at different strain levels. Int. J. Plast..

[16] Bettinali L., Tosti S., Pizzuto A. (2014). Mechanical and Electrical Properties of Cryo-worked Cu. J. Low Temp. Phys..

[17] Wang S., Zhu W., Yu P., Wang X., He T., Tan G., Ning C. (2015). Antibacterial nanostructured copper coatings deposited on tantalum by magnetron sputtering. Mater. Technol..

[18] Momeni M.M., Hashemizadeh S., Mirhosseini M., Kazempour A., Hosseinizadeh S.A. (2016). Preparation, characterisation, hardness and antibacterial properties of Zn–Ni–TiO_2_ nanocomposites coatings. Surf. Eng..

[19] Harris L.G., Foster S.J., Richards R.G. (2002). An introduction to Staphylococcus aureus, and techniques for identifying and quantifying, S. aureus adhesisn in relation to adhesion to biomaterials: Review. Eur. Cells Mater..

[20] Ma N., Cameron A., Tivey D., Grae N., Roberts S., Morris A. (2017). Systematic review of a patient care bundle in reducing staphylococcal infections in cardiac and orthopaedic surgery. ANZ J. Surg..

[21] Chen A.F., Wessel C.B., Rao N. (2013). Staphylococcus aureus screening and decolonization in orthopaedic surgery and reduction of surgical site infections infection. Clin. Orthop. Relat. Res..

[22] Otto M. (2008). Staphylococcal biofilms. Curr. Top. Microbiol. Immunol..

[23] Sivasankaran S., Alaboodi A.S., Al-Mufadi F. (2018). Cold deformation of dezincification resistant yellow brass for plumbing applications. Mater. Manuf. Process..

[24] Domashenkov A., Borbély A., Smurov I. (2017). Structural modifications of WC/Co nanophased and conventional powders processed by selective laser melting. Mater. Manuf. Process..

[25] Gay P., Hirsch P.B.K.A. (1953). The estimation of dislocation densities in metals from X-ray data. Acta. Metall..

[26] Jiang J., Britton T.B., Wilkinson A.J. (2012). Accumulation of geometrically necessary dislocations near grain boundaries in deformed copper. Philos. Mag. Lett..

[27] Hall G.K., Williamsons W.H. (1953). X-ray line broadening from filed aluminum and wolfram. Acta. Metall..

[28] Hordon M.J., Averbach B.L. (1961). X-ray measurement of dislocation density in deformed copper and aluminum single crystals. Acta Metall..

[29] Mishnaevsky L., Levashov E., Valiev R.Z., Segurado J., Sabirov I., Enikeev N., Prokoshkin S., Solov’yov A.V., Korotitskiy A., Gutmanas E. (2014). Nanostructured titanium-based materials for medical implants: Modeling and development. Mater. Sci. Eng. R Rep..

[30] Sunil B.R., Kumar A.A., Sampath Kumar T.S., Chakkingal U. (2013). Role of biomineralization on the degradation of fine grained AZ31 magnesium alloy processed by groove pressing. Mater. Sci. Eng. C.

[31] Vlack L.H.V. (1989). Elements of Materials Science and Engineering.

[32] Gremaud G. (2004). Overview on dislocation-point defect interaction: The brownian picture of dislocation motion. Mater. Sci. Eng. A.

[33] Hirel P., Carrez P., Clouet E., Cordier P. (2016). The electric charge and climb of edge dislocations in perovskite oxides: The case of high-pressure MgSiO_3_ bridgmanite. Acta Mater..

[34] Osetsky Y.N., Bacon D.J., Rong Z., Singh B.N. (2004). Dynamic properties of edge dislocations decorated by interstitial loops in α-iron and copper. Philos. Mag. Lett..

[35] Koehler J.S., Langreth D., Von Turkovich B. (1962). Charged disloctions in ionic crystals. Phys. Rev..

[36] Pödör B. (1966). Electron Mobility in Plastically Deformed Germanium. Phys. Status Solidi.

