# Supplementary material for: Relationship between Dislocation Density and Antibacterial Activity of Cryo-Rolled and Cold-Rolled Copper
Source: Materials (Basel). 2019 Jan 9;12(2):200. doi: 10.3390/ma12020200 (PMC6356488; doi:10.3390/ma12020200)
Supplement: Supplementary file 1 [file materials-12-00200-s001.pdf]

materials

Article

Relationship between Dislocation Density and Antibacterial Activity of Cryo-Rolled and Cold-Rolled Copper

Vinod Parmar [1,2], Kandarp Changela [3], B. Srinivas [4], Manimuthu Mani Sankar [5], Sujata Mohanty [5], S.K. Panigrahi [4], K. Hariharan [4,] and Dinesh Kalyanasundaram [1,6,*]

[1] Centre for Biomedical Engineering, Indian Institute of Technology Delhi, New Delhi 110016, India; vinodparmar.9876@gmail.com (V.P.)
[2] Department of Physics, Indian Institute of Technology Delhi, New Delhi 110016, India
[3] Department of Mechanical Engineering, Indian Institute of Technology Delhi, New Delhi 110016, India; kandarp.changela06@gmail.com (K.C.)
[4] Department of Mechanical Engineering, Indian Institute of Technology Madras, Chennai, 600036 India; srinivasbehera108@gmail.com (B.S.); skpanigrahi@iitm.ac.in (S.K.P.); hariharan@iitm.ac.in (K.H.)
[5] Stem Cell Research Facility, All India Institute of Medical Sciences, New Delhi 110029 India; sankar.mm@gmail.com (M.M.); drmohantysujata@gmail.com (S.M.);
[6] Department of Biomedical Engineering, All India Institute of Medical Sciences, New Delhi, India
* Correspondence: dineshk.iitdelhi@gmail.com (D.K.)

Received: 13 December 2018; Accepted: 03 January 2019; Published: date

S1. Physical and Mechanical Tests

S1.1 Contact Angle Measurements

Contact angle measurements were performed by using DataPhysics OCA 15EC goniometer (DataPhysics Instruments GmbH, Germany). Two-microliter droplets of deionized (DI) water were dispensed on to the surface of the coupons. After one minute of stabilization, the contact angles were measured. Surface of the pristine copper was found to be hydrophobic in nature with an apparent contact angle of ~112.43 ± 0.32°. The apparent contact angle of cold rolled copper was approximately 96.27 ± 1.36° while that of cryo-rolled was found to be 80.80 ± 4.28° (hydrophilic regime) (Figure S1).

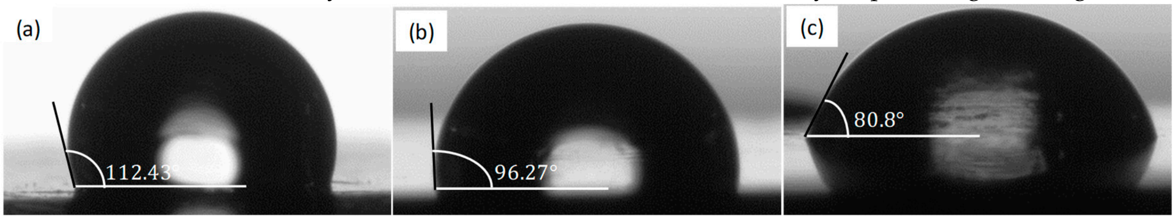

Figure S1. Apparent contact angle (**a**) pristine, (**b**) cold rolled and (**c**) cryo-rolled copper.

S1.2 Results of Tensile Tests

For tensile testing, the substrates were profile cut by wire cut electric discharge machining. The profile dimensions and sample thickness were in accordance to ASTM-ES standard. The substrates were subjected to a tensile load at quasi-static strain rate in a universal tensile testing machine (Model: 5582, Capacity: 50 kN, Instron, USA). The stress–strain plot of cold rolled and cryo-rolled coupons were obtained. Tensile test results indicate that the cryo-rolling process works in such a way that it enhances the strength of material under process. Thus cryo-rolled substrates are able to bear higher stress (~700 MPa) as compare to cold rolled (~480 MPa) and pristine (~320 MPa) copper sample, as shown in Figure S2.

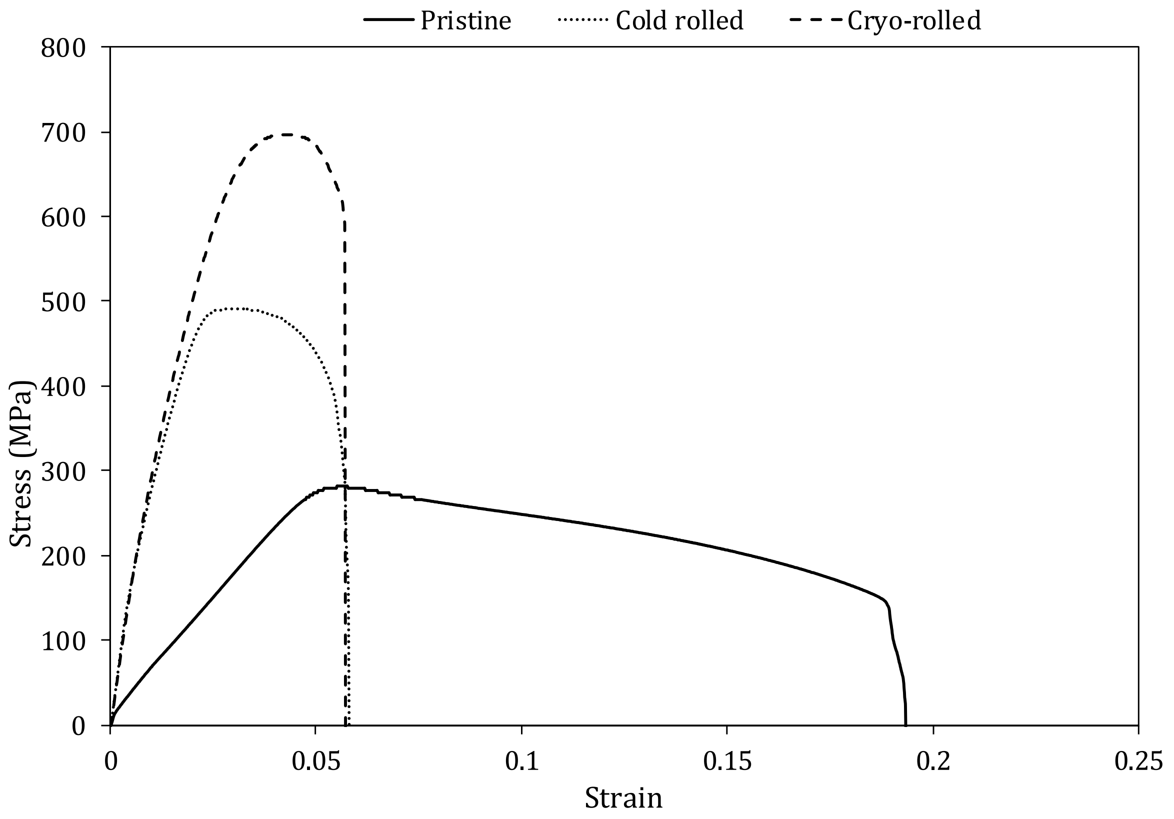

Figure S2. Stress–strain curve depicting tensile test results of pristine, cold rolled, cryo-rolled 1, and cryo-rolled 2 copper substrates.

S1.3 Estimation of Leaching of Copper

The leaching of copper in liquid media (such as nutrient broth) was estimated using inductively coupled plasma-mass spectroscopy (Model: ICP-MS 7900, manufacturer: Agilent, USA). Cleaned and autoclaved coupons of dimension 5 mm × 10 mm × 6 mm were sectioned from pristine copper plate, while coupons of dimension 5 mm X 10 mm X 1 mm were sectioned from cold-rolled and cryo-rolled copper plates. The unrolled pristine coupons are six times thicker than cold-rolled and cryo-rolled coupons. For natural leaching of elemental copper, each of the coupons were immersed in falcon tubes containing 5 mL nutrient broth media for 72 hours at 37 °C. After 72 hours, all the copper coupons were taken out and left out media (with leached copper) was diluted by a factor of 20 to lower the concentration for ICP-MS measurements.

The quantitative results of ICP-MS indicate that 644.5, 975.9, and 1441.5 ppb/mm^2 of elemental copper leached out from pristine, cold rolled and cryo-rolled copper coupons respectively in to the nutrient broth. Therefore, higher concentration of copper leaching has led to higher contact killing *S. aureus* by cryo-rolled copper. Hong et al. has proposed an oxidation-dissolution mechanism that leads to leaching of copper ions in liquid media, wherein the free hydroxyls attack the metal surface to form oxidized product from the surface [1]. The dissolution of copper ions is highly affected by the disinfectant dosage, immersion time and pH of the aqueous media. Of the above parameters, pH is the most significant parameter that determines the solubility of various oxidation states of copper (e.g., cupric oxide (CuO) or copper oxide (Cu_2O_3) state). Low pH media will readily dissolve the copper mineral (cupric oxide) and thus results in increased carbonation and exposes fresh copper metal sites to further oxidation and leaching.

S2. Biological Tests

S2.1 Estimation of leakage protein

Leakage of endo-cell proteins were determined by spectrophotometry, as described elsewhere [2]. *S. aureus* was grown for 12 hours in 100 mL of Luria broth (Hi-Media, India) at 37 °C. The cells were pelleted at 10,000 g for 10 minutes and washed twice with 1X PBS (pH 6.8). The pellet was re-suspended in 50 mM sodium phosphate buffer (pH 7.1) and turbidity was adjusted to 1 OD. The cell suspension was seeded on to the surface of copper coupons followed by incubation for 24 hours at 37 °C. For positive controls, *S. aureus* suspension was treated with 10 µg/mL cetyl trimethylammonium bromide at 37 °C for 120 minutes, followed by bath sonication for 15 minutes. The suspension was centrifuged at 10,000 g for 10 minutes. The absorbance of the supernatant at 260- and 280- nm was determined using spectrophotometer (BioTek, USA). Protein concentrations in the leaked supernatants was estimated as described elsewhere [3]. Protein leakage from bacterial suspensions not exposed to copper coupons served as negative control. The extent of leakage of 260- and 280- nm absorbing compounds were expressed as a percentage of the positive control (suspension treated with CTAB) measured in the supernatant.

Based on the bacterial killing of copper coupons, we investigated the exact mechanism of action. The membrane leakage assay demonstrated damage caused to the cytoplasmic membrane of *S. aureus* when exposed to copper coupons. The 260- and 280-nm absorbing material released in the supernatants were measured at 0 and 24 hours after exposure (Figure S3). Leakage of proteins from all the tested coupons were similar at 0 hours. Post 24 hours exposure of *S. aureus* cells, cryo-rolled induced higher leakage of proteins (twice) as compared to pristine and cold rolled copper coupons. This indicates the disruption of the cell membrane of *S. aureus*.

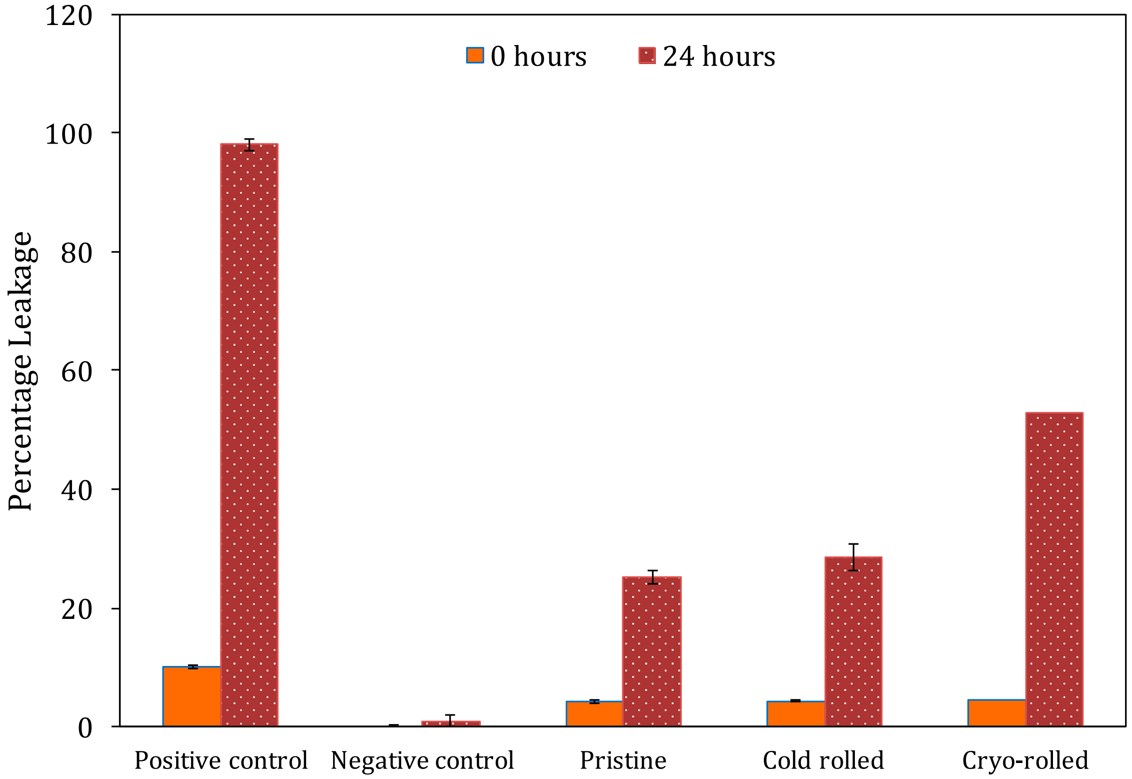

Figure S3. Percentage leakage of protein exposed to copper coupons.

S2.2 Ethidium Bromide (EtBr) Uptake Assay

The disruptive effect of copper coupons on *S. aureus* was assessed by EtBr uptake. One milliliter of 1 × 10^6 CFU/mL cell suspension of *S. aureus* in 1X PBS (pH 6.8) was incubated on copper coupons at 37 °C for 24 hours. Post incubation, adherent *S. aureus* were retrieved as described in the main script. EtBr (1 mg/mL solution) was added to all copper exposed *S. aureus* cells and incubated at room temperature for 15 minutes. *S. aureus* treated with 1% Triton X-100 was used as positive control whereas cells with EtBr not exposed to copper substrates served as negative controls. Ten microliters

of each suspension were examined under a fluorescence microscope at 100X magnification (Nikon, Japan) for fluorescence. EtBr is a DNA intercalating agent which enters the cell wall and binds with DNA of the damaged cell and thus results in the emission of higher fluorescence. *S. aureus* cellular damage by copper coupons were estimated by EtBr uptake and visualized by fluorescent microscopy. *S. aureus* cells exposed to cryo-rolled coupons exhibited increased staining with EtBr as compared to pristine and cold rolled copper (Figure S4). EtBr uptake was conducted to measure the cell integrity of *S. aureus* when exposed to copper coupons. The significant number of fluorescence spots on cryo-rolled copper represents nearly complete cell membrane rupture (Figure S4c,d).

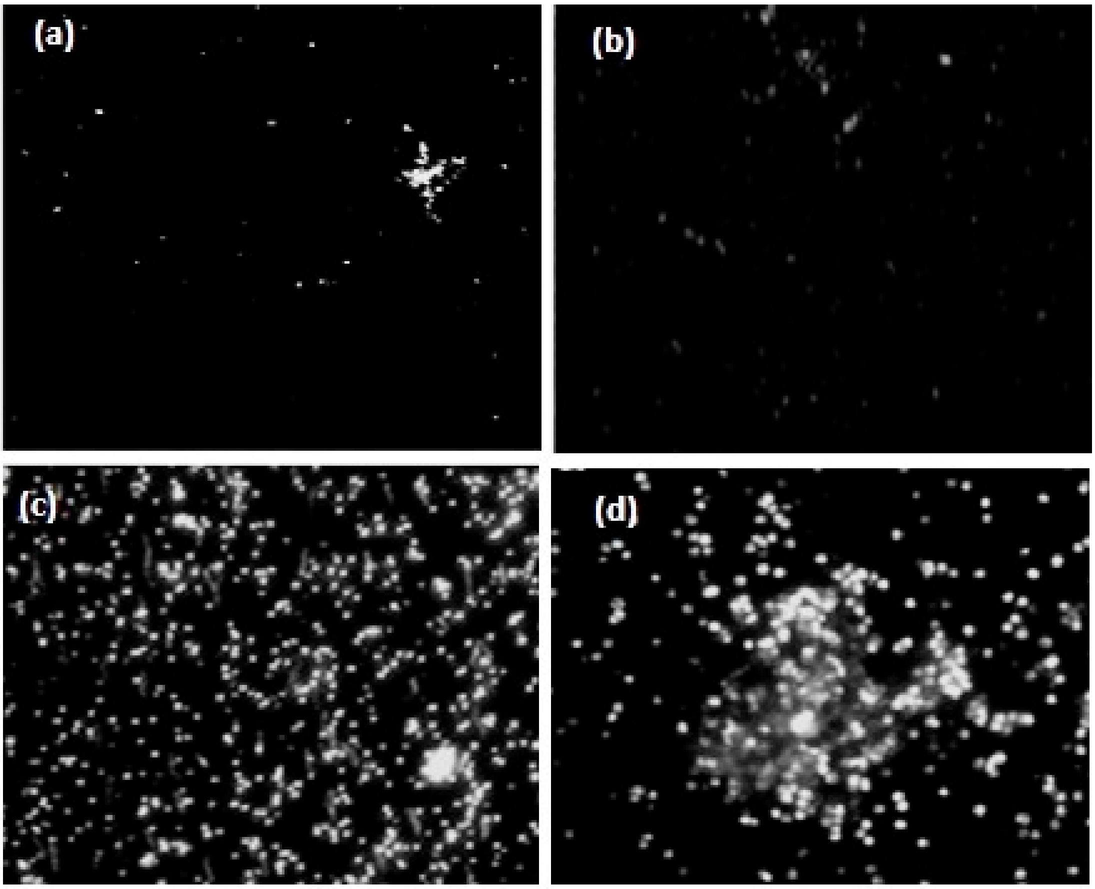

Figure S4. EtBr uptake of *S. aureus* on (**a**) pristine, (**b**) cold rolled, (**c**) cryo-rolled copper site1, and (**d**) cryo-rolled copper site 2 coupons (100X magnification).

S2.3 Hemolysis Experiment for Non-Toxicity of Copper Coupons

The toxicity assessment of copper coupons were tested for hemolysis [4]. This study was approved by the Institute Ethics Committee, All India Institute of Medical Sciences, New Delhi. Human peripheral blood samples were collected from healthy volunteers in EDTA vacutainer tubes after obtaining informed consent. Whole blood (5 mL) was added to 10 mL of 1X PBS (pH 6.8) and red blood cells (RBCs) were separated by centrifuging at 10,000 g for 10 minutes. The pelleted RBCs were washed thrice with 10 mL of 1X PBS (pH 6.8) and diluted to 50 mL with 1X PBS. Two hundred microliters of the diluted RBCs were layered onto the surface of copper coupons. RBCs treated with 1% Triton X-100 was used as a positive control, and RBCs not exposed to copper coupons served as a negative control. The copper coupons were further incubated at 37 °C for 3 hours. After incubation, the resultant suspension was aspirated and centrifuged at 10,000 g for 3 minutes. The hemoglobin absorbance of the supernatant was measured at 570 nm using a spectrophotometer (BioTek, USA). The tests were performed in triplicates. No cytotoxicity was observed on any of the copper coupons and no significant differences were observed between the tested copper coupons. This provides

evidence of the non-toxic nature of copper to human cells. Physical alteration of pristine copper does not alter the non-toxic nature of copper after cryo-rolling or cold rolling.

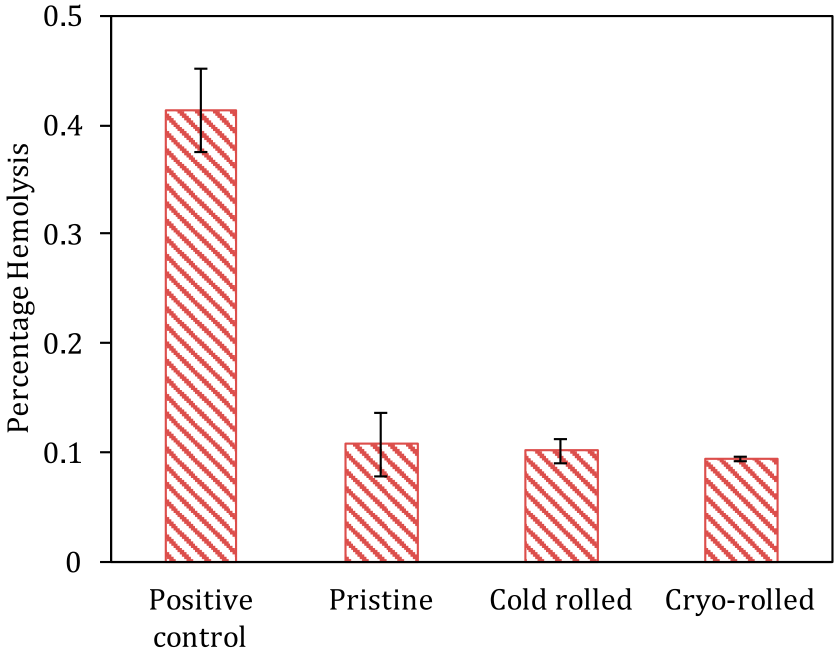

Figure S5. Percentage hemolysis of RBCs exposed to copper coupons.

References

1. Hong, P. & Macauley, Y. Corrosion and leaching of copper tubing exposed to chlorinated drinking water. *Water. Air. Soil Pollut.* **1998**,*108*, 457–471.
2. Cox, S. D.; Mann,C.M.; Markham, J.L.; Gustafson, J.E.; Warmington, J.R.; Wyllie, S.G. Determining the antimicrobial actions of tea tree oil. in *Molecules* **2001**, *6*, 87–91.
3. LOWRY, O. H., ROSEBROUGH, N. J., FARR, A. L. & RANDALL, R. J. Protein measurement with the Folin phenol reagent. *J. Biol. Chem.* **1951**, *193*, 265–275.
4. Laloy, J.; Minet, V.; Alpan, L.; Mullier, F.; Beken, S.; Toussaint, O.; Lucas, S.; Dognél, J.M. Impact of Silver Nanoparticles on Haemolysis, Platelet Function and Coagulation. *Nanobiomedicine* **2014**, *1*, 1–9.
